# Additional prognostic value of toe-brachial index beyond ankle-brachial index in hemodialysis patients

**DOI:** 10.1186/s12882-020-01991-7

**Published:** 2020-08-20

**Authors:** Manabu Hishida, Takahiro Imaizumi, Steven Menez, Masaki Okazaki, Shin’ichi Akiyama, Hirotake Kasuga, Junichi Ishigami, Shoichi Maruyama, Kunihiro Matsushita

**Affiliations:** 1 Department of Epidemiology, Johns Hopkins Bloomberg School of Public Health, 2024 E. Monument Street, Suite 2-600, Baltimore, MD 21287 USA; 2 Department of Nephrology, Graduate School of Medicine, Nagoya University, Nagoya, Japan; 3 Division of Nephrology, Department of Medicine, Johns Hopkins University School of Medicine, Baltimore, MD 21287 USA; 4 Kaikoukai Healthcare Group Kaikoukai Central Clinic, Nagoya, Japan

**Keywords:** Hemodialysis, Peripheral artery disease, Mortality, Ankle brachial index, Toe brachial index

## Abstract

**Background:**

Ankle-brachial index (ABI), the first-line diagnostic test for peripheral artery disease, can be falsely elevated when ankle arteries are incompressible, showing a J-shaped association with mortality. In this situation, toe-brachial index (TBI) is the recommended test. However, whether TBI provides additional prognostic information beyond ABI in patients on hemodialysis is unknown.

**Methods:**

In this retrospective cohort study of 247 Japanese prevalent hemodialysis patients (mean age 66.8 [SD 11.6] years), we evaluated mortality (116 deaths over a median follow-up of 5.2 years) related to quartiles of ABI and TBI, as well as three categories of low ABI (≤0.9), normal/high ABI (> 0.9) + low TBI (≤0.6), and normal/high ABI + normal TBI (> 0.6) using multivariable Cox models.

**Results:**

ABI showed a J-shaped association with mortality (adjusted hazard ratio 2.72 [95% CI, 1.52–4.88] in the lowest quartile and 1.59 [95% CI, 0.87–2.90] in the highest quartile vs. the second highest). Lower TBI showed a potentially dose-response association with mortality (e.g., adjusted hazard ratios 2.63 [95% CI, 1.36–5.12] and 2.89 [95% CI, 1.49–5.61] in the lowest two quartiles vs. the highest). When three categories by both ABI and TBI were analyzed, those with low ABI (≤0.9) experienced the highest risk followed by normal/high ABI (> 0.9) + low TBI (≤0.6). Among patients with normal/high ABI (> 0.9), the increased mortality risk in individuals with low TBI (≤0.6) compared to those with normal TBI (> 0.6) were significant (adjusted hazard ratio 1.84 [95% CI, 1.12–3.02]).

**Conclusions:**

Lower TBI was independently associated with mortality in patients on hemodialysis and has the potential to classify mortality risk in patients with normal/high ABI. Our results support the importance of evaluating TBI in addition to ABI in this clinical population.

## Background

Lower-extremity peripheral artery disease (PAD) is recognized as a major risk factor for amputation, myocardial infarction, and stroke [1, 2]. Patients with chronic kidney disease (CKD) are prone to developing PAD [3–5]. This is especially true in patients on hemodialysis. Indeed, the incidence of PAD is higher than that of myocardial infarction or stroke in this clinical population [6].

Major guidelines recommend the ankle-brachial index (ABI) as the first-line non-invasive diagnostic test for PAD [2, 7, 8]. However, ABI may be falsely high in individuals with calcified ankle arteries [9, 10]. Indeed, previous studies demonstrated a J-shaped association between ABI and mortality (i.e., elevated risk in both lower and higher ABI) [11, 12], which may complicate the interpretation of ABI, especially in individiuals prone to vascular calcification like patients on hemodialysis and diabetes mellitus [9, 10, 13–15].

To overcome this caveat of ABI, clinical guidelines recommend using toe-brachial index (TBI) when ABI is greater than 1.4, since toe arteries are less likely to be calcified [16, 17]. Importantly, a few studies have reported that TBI provides additional or even better prognostic information than ABI in persons with diabetes [18] and CKD [19]. A similar result was recently reported among patients on hemodialysis [20]. However, this study included only 37 patients, and thus a larger comprehensive study is necessary.

Therefore, we sought to quantify and contrast the association of ABI and TBI with mortality in patients on hemodialysis. Additionally, we assessed whether TBI values could distinguish the risk of mortality in patients with normal/high ABI.

## Materials and methods

### Study participants

We conducted a retrospective cohort study using data from Kaikoukai Central Clinic, which is one of several Kaikoukai Healthcare Group outpatient hemodialysis facilities in Japan. Between April 2009 and July 2015 (baseline period), we identified 659 consecutive patients aged 20 years or older who received maintenance hemodialysis therapy. For the current study, we investigated 247 prevalent hemodialysis patients with data on both ABI and TBI measurements prior to the baseline date described below (Supplementary File: Fig. S1). Patients excluded from this study had generally similar characteristics to the study population in terms of age, gender, and the prevalence of hypertension. The major differences were seen in hemodialysis vintage, a history of cardiovascular disease (CVD), and primary kidney disease (Supplementary File: Table S1).

### Baseline variables

For patients who were already receiving hemodialysis at this clinic at the start of the baseline period, the baseline date for observation was set to April 1st, 2009 (*n* = 239). For those patients who were referred to this clinic for hemodialysis maintenance during the baseline period, the baseline date was set to the day of referral (*n* = 8). Age, gender, hemodialysis vintage, smoking status, primary kidney disease, history of PAD and other CVD (coronary artery disease, heart failure, and stroke), other medical comorbidities, and home medications at the baseline were systematically abstracted from medical records.

We used laboratory data collected most closely to the baseline, at a median of 6 (inter quartile interval [IQI] 5–12]) days after baseline. For those who had a hemodialysis vintage of < 3 months at baseline (*n* = 2), we used blood tests collected three months after hemodialysis initiation. This approach was based on the fact that laboratory variables might be unstable in the first few months after hemodialysis initiation, as previously reported [21, 22].

The ABI and TBI measurements were performed according to clinical indications (e.g., intermittent claudication and coldness of feet). ABI and TBI were obtained at any time during a patient’s hemodialysis session in a room maintained at a temperature between 26 and 27 °C, using a non-invasive oscillometric device (BP-203RPE III system [OMRON HEALTHCARE Co., Ltd., Kyoto, Japan]), with the patient in the supine position. Blood pressure cuffs were placed on the arm without the arteriovenous fistula or graft, on the ankles, and at the bases of the big toes. ABI and TBI were determined as the ratio of ankle systolic blood pressure and toe systolic blood pressure to brachial systolic blood pressure, respectively. ABI and TBI were measured bilaterally on the same day, and the lower values of right and left ABI and TBI were used for the analysis.

### Outcomes

The primary outcome was all-cause mortality, and we also secondarily investigated CVD mortality and non-CVD mortality, separately. CVD mortality was defined as death due to coronary artery disease, stroke, heart failure, aortic disease, or sudden cardiac death. Outcome data were available as long as the patients were followed within the Kaikoukai Healthcare Group. Patients were followed until death (*n* = 116), loss to follow-up (*n* = 30), or the end of follow-up (*n* = 101) on 31 March 2016 (i.e., administrative censoring).

### Statistical analysis

Baseline characteristics were expressed as mean (SD) or number (percentage). One-way ANOVA with post-hoc Tukey test was used to compare the characteristics across TBI quartiles. We first estimated survival across ABI and TBI quartiles using the Kaplan-Meier method and assessed the difference in survival estimates by the log-rank test.

For the primary analysis, we imputed missing data on some covariates (i.e., systolic blood pressure [missing in *n* = 2], diabetes mellitus [*n* = 6], calcium [*n* = 8], phosphate [n = 8], total cholesterol [*n* = 29], hemoglobin [*n* = 23], albumin [*n* = 34], and smoking status [*n* = 35]) using multiple imputation by chained equations. In brief, missing values were imputed and regressed on all variables in the analysis, including outcome variables. Each imputation was conducted by logistic regression for binary variables, and by linear regression for continuous variables. This procedure was then repeated 25 times to construct a pooled dataset [23].

Using the imputed data set, multivariable Cox models were conducted to estimate the hazard ratios (HRs) of all-cause mortality, CVD mortality, and non-CVD mortality by ABI and TBI quartiles. We explored four models: Model 1 was unadjusted; Model 2 adjusted for age and gender; Model 3 additionally adjusted for a clinical history of diabetes, smoking status, CVD history (PAD and other CVD), and hemodialysis vintage; and Model 4 further adjusted for systolic blood pressure, total cholesterol, hemoglobin, serum albumin, serum calcium, and serum phosphate. We selected the second highest quartile of ABI and the highest quartile of TBI as a reference based on their respective J-shaped and linear relationships to mortality from previous literature [11, 12, 18]. P for trend was calculated in the lowest three quartiles for ABI (due to its J-shaped association) and in all quartiles for TBI by modeling them as continuous variables.

Subsequently, we assessed the mortality risk across categories using combinations of ABI and TBI. Since there were few patients with low ABI but normal TBI, we investigated the following three categories: Low ABI, normal/high ABI + low TBI, and normal/high ABI + normal TBI. According to the clinical guidelines, low ABI was defined as a value ≤0.9 [16, 17]. For TBI, based on the guidelines we primarily explored a TBI cutoff of ≤0.7 [2, 24]. However, given limited evidence behind this threshold, we secondarily investigated a cutoff of ≤0.6 based on prior literature [25].

We conducted a few sensitivity analyses. First, we stratified by several pre-specified demographic and clinical factors: age, gender, body mass index (BMI) (< vs. ≥22 kg/m^2^), diabetes status, and systolic blood pressure (< vs. ≥130 mmHg). The interactions of ABI and TBI categories with stratifying factors were evaluated by likelihood-ratio testing. Next, we restricted our analysis to 193 patients who had ABI and TBI measurements within two years prior to baseline, to minimize their misclassification due to changes over time. We also performed complete case analysis including all 247 patients in Models 1 and 2, 212 patients in Model 3, and 176 patients in Model 4. In all analyses, a *p*-value less than 0.05 was considered statistically significant. All statistical analyses were performed using Stata version 14 (Stata Corp, College Station, Texas, USA).

## Results

### Baseline characteristics

**Fig. 1:**
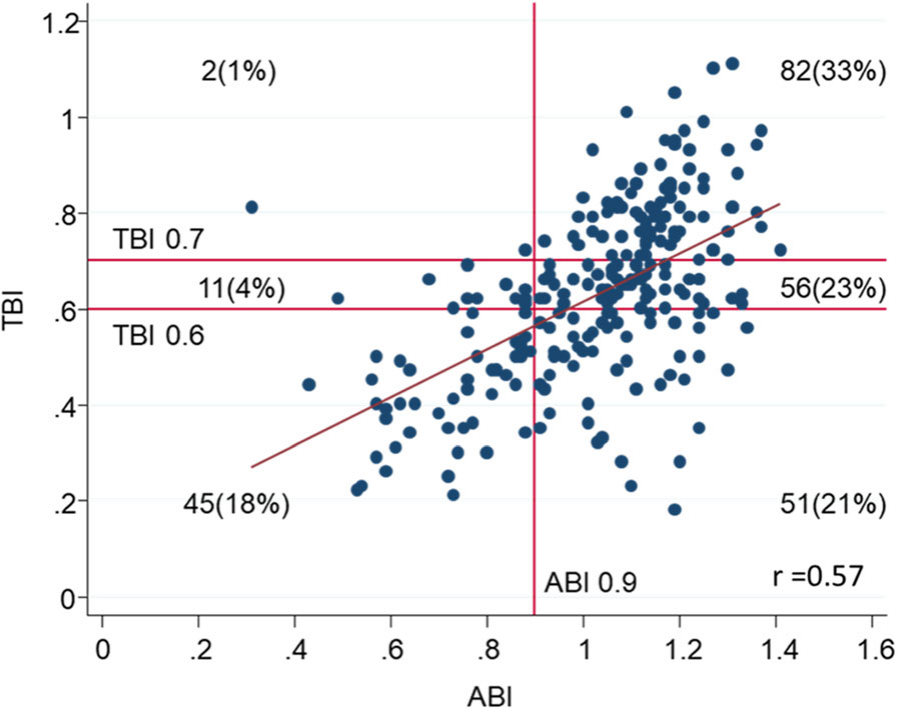
Distribution and correlation of ABI and TBI

The mean age of the 247 patients was 66.8 (SD 11.6) years, and 167 were men (67.6%). The median ABI and TBI were 1.07 (IQI 0.91–1.17) and 0.63 (0.5–0.76), respectively. The median elapsed time between ABI/TBI measurement and baseline was 0.69 (IQI 0.33–1.54) years. As shown in Fig. 1, ABI and TBI showed a moderate correlation (r = 0.57). Low ABI (≤0.9) was observed in 58 patients (23.5%), whereas TBI ≤0.7 and ≤ 0.6 were observed in 163 (66.0%) and 96 (38.9%) patients, respectively. Patients with lower TBI were more likely to be older, have diabetes, and have a history of PAD and other CVD (Table 1). In contrast, there were no evident differences in BMI or hypertension. Patients with lower ABI were also likely to be older, have diabetes, and have a history of CVD (Supplementary File: Table S2).

**Table 1:** Baseline characteristics of patients stratified by TBI quartile

Characteristic	Lower TBI (Quartile)					*P*-value	Post hoc
	Q1 (0.18–0.50)	Q2 (0.51–0.63)	Q3 (0.64–0.76)	Q4 (0.77–1.11)	Total		
	(*n* = 63)	(*n* = 61)	(*n* = 65)	(*n* = 58)	(*n* = 247)		
Age (years)	70.2 ± 11.2	68.6 ± 11.2	66.6 ± 10.2	61.5 ± 12.4	66.8 ± 11.6	< 0.01	c, e
Hemodialysis vintage (year)	7.6 ± 6.9	9.2 ± 8.7	11.3 ± 8.9	9.9 ± 7.9	9.5 ± 8.2	0.08	
Gender (male) (%)	46 (73%)	37 (61%)	42 (65%)	42 (72%)	167 (68%)	0.385	
Body mass index (kg/m^2^)	21.8 ± 3.6	22.3 ± 3.2	22.1 ± 3.9	22.0 ± 3.8	22.1 ± 3.6	0.891	
History of smoking							
Ever (%)	11 (17%)	11 (18%)	13 (20%)	16 (28%)	51 (21%)	0.504	
Never (%)	42 (67%)	39 (64%)	46 (71%)	34 (59%)	161 (65%)	0.557	
Unknown (%)	10 (16%)	11 (18%)	6 (9%)	8 (14%)	35 (14%)	0.534	
Comorbidity							
Diabetes mellitus (%)	44 (71%)	31 (52%)	22 (35%)	18 (32%)	115 (48%)	< 0.01	b, c
Hypertension (%)	40 (69%)	45 (78%)	44 (72%)	44 (81%)	173 (75%)	0.427	
Lipid metabolism disorder (%)	9 (16%)	9 (16%)	4 (7%)	4 (8%)	26 (12%)	0.246	
PAD (%)	55 (87%)	46 (75%)	49 (75%)	24 (41%)	174 (70%)	< 0.01	c, e, f
Other CVDs (CAD, HF, stroke) (%)	51 (81%)	41 (67%)	39 (60%)	31 (53%)	162 (66%)	< 0.01	c
Primary kidney disease							
Diabetic nephropathy (%)	43 (68%)	31 (51%)	20 (31%)	17 (29%)	111 (45%)	< 0.01	b, c
Nephrosclerosis (%)	2 (3%)	3 (5%)	5 (8%)	6 (10%)	16 (6%)	0.4	
Chronic glomerulonephritis (%)	12 (19%)	13 (21%)	23 (35%)	17 (29%)	65 (26%)	0.138	
Polycystic kidney disease (%)	2 (3%)	2 (3%)	3 (5%)	3 (5%)	10 (4%)	0.929	
Other (%)	1 (2%)	7 (11%)	9 (14%)	6 (10%)	23 (9%)	0.092	
Unknown (%)	3 (5%)	5 (8%)	5 (8%)	9 (16%)	22 (9%)	0.204	
Systolic blood pressure (mmHg)	150 ± 22	151 ± 25	146 ± 22	147 ± 23	149 ± 23	0.551	
Diastolic blood pressure (mmHg)	77 ± 11	79 ± 11	82 ± 11	85 ± 14	81 ± 12	< 0.01	c, e
Total cholesterol (mg/dl)	163 ± 38	154 ± 32	155 ± 30	150 ± 33	156 ± 33	0.185	
Oral medication							
Antihypertensive drugs (%)	37 (59%)	36 (59%)	35 (54%)	41 (71%)	149 (60%)	0.281	
Lipid-lowering drugs (%)	2 (3%)	4 (7%)	1 (2%)	1 (2%)	8 (3%)	0.37	
Hemoglobin (g/dl)	10.6 ± 1.2	10.7 ± 1.3	10.6 ± 1.3	10.8 ± 1.4	10.7 ± 1.3	0.891	
Albumin (g/dl)	3.7 ± 0.4	3.6 ± 0.3	3.7 ± 0.3	3.8 ± 0.3	3.7 ± 0.3	0.231	
Calcium (mg/dl)	8.5 ± 0.6	8.7 ± 0.6	8.9 ± 0.7	9.0 ± 0.7	8.8 ± 0.7	< 0.01	b, c, e
Phosphate (mg/dl)	5.0 ± 1.6	4.8 ± 1.4	4.8 ± 1.3	5.7 ± 1.3	5.1 ± 1.4	< 0.01	e, f
ABI	0.85 ± 0.22	1.02 ± 0.17	1.09 ± 0.13	1.16 ± 0.15	1.03 ± 0.20	< 0.01	a, b, c, e

Values are mean ± SD, % of the total. Missing values: Body mass index (n = 3), History of smoking (n = 35), Diabetes mellitus (n = 6), Hypertension (*n* = 16), Lipid metabolism disorder (*n* = 21), Systolic blood pressure (n = 2), Total cholesterol (*n* = 29), Hemoglobin (n = 23), Albumin (*n* = 34), Calcium (n = 8), and Phosphate (n = 8). PAD peripheral artery disease, CVD cardiovascular disease, CAD coronary artery disease, HF heart failure, ABI ankle-brachial index ^a^significant difference between Q1 vs. Q2; ^b^significant difference between Q1 vs. Q3; ^c^significant difference between Q1 vs. Q4; ^d^significant difference between Q2 vs. Q3; ^e^significant difference between Q2 vs. Q4; ^f^significant difference between Q3 vs. Q4

### Independent associations of ABI and TBI with mortality

**Fig. 2:**
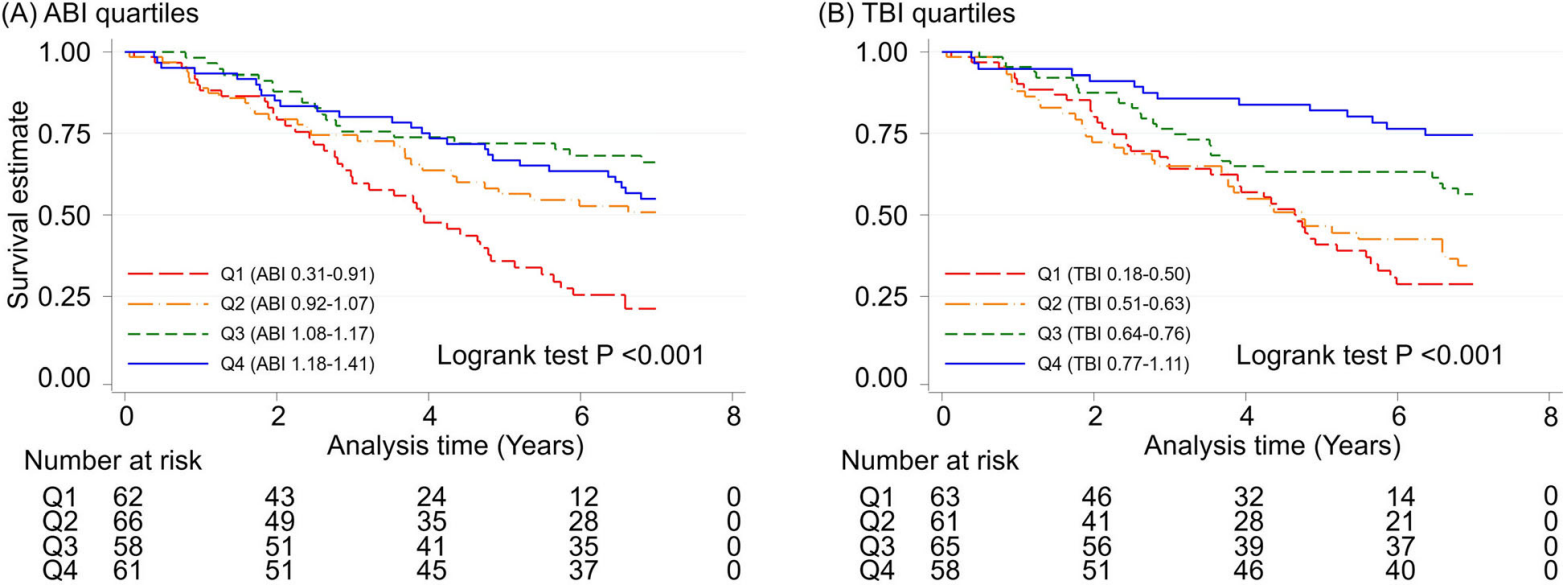
Survival estimates (all-cause mortality) according to ABI and TBI quartiles

During a median follow-up of 5.2 (IQI 2.4–7.0) years, there were 116 deaths (47.0%). Figure 2 shows the Kaplan-Meier curves for all-cause mortality according to ABI quartiles (Fig. 2a) and TBI quartiles (Fig. 2b). As expected, ABI showed a J-shaped pattern for all-cause mortality, with the lowest ABI quartile (ABI 0.31–0.91) having the worst prognosis and the highest quartile (ABI 1.18–1.41) demonstrating slightly worse prognosis than the second highest quartile (ABI 1.08–1.17). In contrast, lower TBI levels showed a potentially dose-response relationship to all-cause mortality, with its lowest quartile (TBI 0.18–0.50) showing the worst prognosis and the highest quartile (TBI 0.77–1.11) having the best prognosis. Similar results were found for both CVD mortality and non-CVD mortality (Supplementary File: Fig. S2).

Elevated mortality risk in the lowest ABI quartile, compared to second highest quartile, remained significant even after adjusting for demographic (Model 2) and clinical (Models 3 and 4) factors (Table 2). For example, the adjusted HR in Model 4 was 2.72 (95% CI, 1.52–4.88). Although not significant, the highest quartile consistently demonstrated a HR > 1 in all models. In contrast, there was largely a dose-response association between TBI and mortality risk. In Model 1, even the second highest quartile with TBI 0.64–0.76 had significantly higher HR compared to the highest quartile (1.98 [95% CI, 1.04–3.78]). The lowest and second lowest quartiles demonstrated significant and similar HRs across all four models (e.g., adjusted HR 2.63 [95% CI, 1.36–5.12] and 2.89 [95% CI, 1.49–5.61], respectively, in Model 4). The results were largely consistent for CVD mortality and non-CVD mortality (Supplementary File: Table S3), although HRs appeared greater for CVD mortality than non-CVD mortality for both ABI and TBI. The exclusion of 12 patients with ABI ≥1.4 did not materially alter the results (Supplementary File: Table S4).

**Table 2:** HRs (95% CI) of all-cause mortality (116 cases) according to ABI/TBI quartile

ABI	Q1 (0.31–0.91)	Q2 (0.92–1.07)	Q3 (1.08–1.17)	Q4 (1.18–1.41)	P for trend (Q1–3)
All-cause mortality	41 deaths / 62	29 deaths / 66	19 deaths / 58	27 deaths / 61	(*n* = 186)
Model 1	3.15 (1.82–5.44) **	1.67 (0.94–2.98)	ref	1.36 (0.76–2.45)	< 0.001
Model 2	2.99 (1.72–5.19) **	1.69 (0.94–3.01)	ref	1.62 (0.90–2.93)	< 0.001
Model 3	2.83 (1.60–5.03) **	1.54 (0.86–2.78)	ref	1.63 (0.90–2.94)	< 0.001
Model 4	2.72 (1.52–4.88) **	1.45 (0.79–2.64)	ref	1.59 (0.87–2.90)	< 0.001
TBI	Q1 (0.18–0.50)	Q2 (0.51–0.63)	Q3 (0.64–0.76)	Q4 (0.77–1.11)	P for trend (Q1–4)
All-cause mortality	40 deaths / 63	35 deaths / 61	27 deaths / 65	14 deaths / 58	(n = 247)
Model 1	3.91 (2.12–7.21) **	3.60 (1.93–6.70) **	1.98 (1.04–3.78) *	ref	< 0.001
Model 2	2.74 (1.47–5.11) **	3.01 (1.61–5.63) **	1.56 (0.81–2.99)	ref	0.001
Model 3	2.54 (1.31–4.94) **	2.80 (1.44–5.45) **	1.43 (0.72–2.86)	ref	0.004
Model 4	2.63 (1.36–5.12) **	2.89 (1.49–5.61) **	1.54 (0.78–3.04)	ref	0.004

Model 1; unadjusted

Model 2; adjusted for age and gender

Model 3; Model 2+ diabetes mellitus, smoking status, history of cardiovascular disease, and hemodialysis vintage

Model 4; Model 3+ systolic blood pressure, total cholesterol, hemoglobin, albumin, calcium, and phosphate

* *P* < 0.05, ** *P* < 0.01

### Combined associations of ABI and TBI with mortality

With TBI 0.7 as a threshold, there were 82 patients with normal/high ABI + normal TBI and 107 patients with normal/high ABI + low TBI. Corresponding numbers were 138 and 51 patients, respectively, when we used TBI 0.6 as a cutoff. Low ABI (≤0.9) was seen in 58 patients, as noted above.

**Fig. 3:**
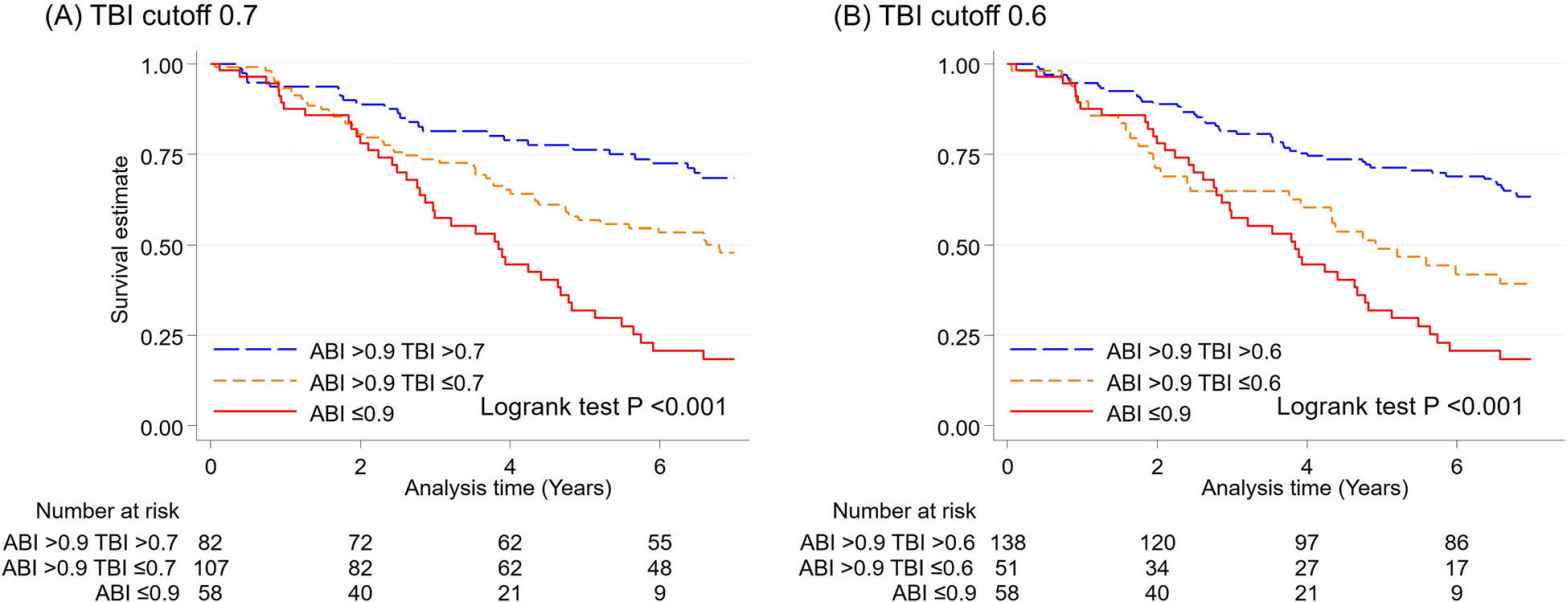
Survival estimates (all-cause mortality) according to three categories of ABI/TBI

Figure 3 shows Kaplan-Meier curves for all-cause mortality according to the three categories of ABI/TBI group, using TBI cutoffs of 0.7 (Fig. 3a) and 0.6 (Fig. 3b). The low ABI category (≤0.9) (red line) showed the worst prognosis. Comparing the two TBI categories with normal/high ABI, low TBI (orange line) showed worse prognosis than normal TBI (blue line), regardless of TBI threshold, although the separation of the two categories appeared more evident with TBI 0.6 as a threshold.

When we contrasted these three categories using Cox models, the low ABI category (≤0.9) consistently demonstrated the highest HR in all models (Table 3). In those with normal/high ABI, low TBI showed significantly higher mortality compared to normal TBI in Model 1 regardless of TBI threshold (HR 1.94 [95% CI, 1.20–3.13] with TBI 0.7 and 2.13 [95% CI, 1.34–3.41] with TBI 0.6). However, the association was attenuated and no longer significant in Models 2–4 when TBI 0.7 was used as a threshold. In contrast, when low TBI was defined as ≤0.6, normal/high ABI + low TBI had significantly higher mortality than normal/high ABI + normal TBI (HR 1.84 [95% CI, 1.12–3.02] in Model 4). Consistent results were observed for CVD mortality and non-CVD mortality (Supplementary File: Fig. S3 and Table S5). Again, the results were largely similar after the exclusion of ABI ≥1.4 (Supplementary File: Table S6).

**Table 3:** HRs (95% CI) of all-cause mortality (116 cases) according to three categories of ABI/TBI

	ABI > 0.9 TBI > 0.7	ABI > 0.9 TBI ≤0.7	ABI ≤0.9
All-cause mortality	25 deaths / 82	51 deaths / 107	40 deaths / 58
Model 1	ref	1.94 (1.20–3.13) **	3.84 (2.32–6.37) **
Model 2	ref	1.60 (0.99–2.61)	2.89 (1.72–4.86) **
Model 3	ref	1.48 (0.89–2.47)	2.75 (1.57–4.82) **
Model 4	ref	1.65 (0.98–2.77)	2.88 (1.62–5.12) **
	ABI > 0.9 TBI > 0.6	ABI > 0.9 TBI ≤0.6	ABI ≤0.9
All-cause mortality	48 deaths / 138	28 deaths / 51	40 deaths / 58
Model 1	ref	2.13 (1.34–3.41) **	3.23 (2.11–4.95) **
Model 2	ref	1.97 (1.23–3.15) **	2.64 (1.71–4.08) **
Model 3	ref	1.89 (1.16–3.06) *	2.63 (1.65–4.17) **
Model 4	ref	1.84 (1.12–3.02) *	2.53 (1.56–4.11) **

Model 1; unadjusted

Model 2; adjusted for age and gender

Model 3; Model 2+ diabetes mellitus, smoking status, history of cardiovascular disease, and hemodialysis vintage

Model 4; Model 3+ systolic blood pressure, total cholesterol, hemoglobin, albumin, calcium, and phosphate

* *P* < 0.05, ** *P* < 0.01

### Sensitivity analysis

**Fig. 4:**
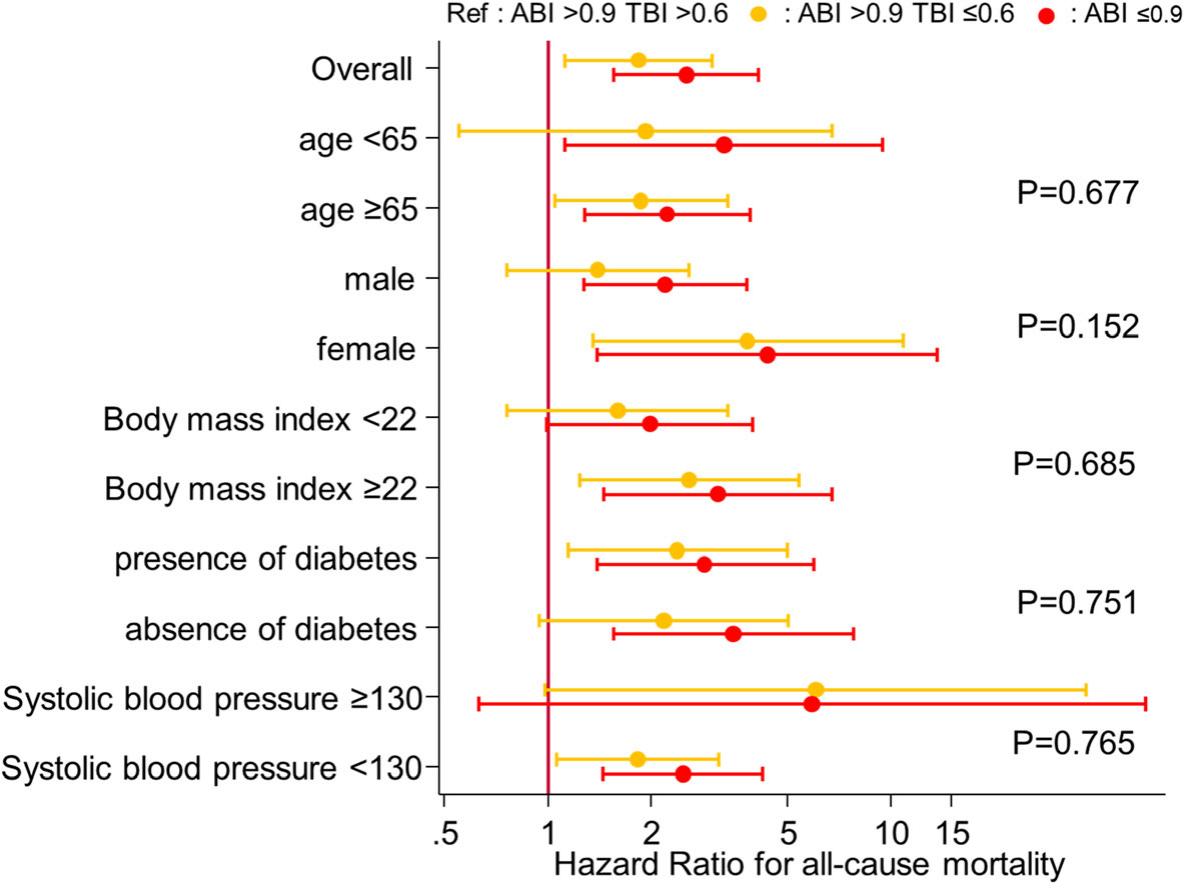
HR (95% CI) of all-cause mortality according to “normal” or “low” ABI/TBI in subgroups. Adjusted for age, gender, diabetes mellitus, smoking status, history of cardiovascular disease, hemodialysis vintage, systolic blood pressure, total cholesterol, hemoglobin, albumin, calcium, and phosphate. The *P* values represent significance levels for interaction terms

The higher mortality risk for normal/high ABI + low TBI vs. normal/high ABI + normal TBI was mostly consistent across demographics and clinical subgroups tested (Fig. 4). The association was prominent in some subgroups (e.g., adjusted HR 3.82 [95% CI, 1.35–10.86] in female and 2.58 [95% CI, 1.24–5.39] in BMI ≥22), although we did not observe statistically significant interactions. In 193 patients who had ABI and TBI measurements within two years prior to baseline, our findings were consistent (Supplementary File: Table S7 and S8). Finally, the results based on complete case analysis were similar to our main analysis (Supplementary File: Table S9 and S10).

## Discussion

Among patients on hemodialysis, we confirmed a J-shaped association between ABI and mortality. In contrast, there was a potential dose-response relationship between TBI and mortality. These associations remained significant even after accounting for several potential confounders, with HRs of all-cause mortality for the lowest ABI and TBI quartiles (vs. respective reference category) ranging from ~ 2 to ~ 4 depending on models. Among patients with normal/high ABI, low TBI vs. normal TBI demonstrated poorer prognosis, with an unadjusted HR ~ 2. This pattern remained consistent even after accounting for potential confounders when TBI 0.6 was used as a threshold of low TBI. These results were largely consistent when we explored CVD and non-CVD mortality as outcomes. The elevated mortality risk in low TBI vs. normal TBI among those with normal/high ABI was consistent across several subgroups, although the association was especially evident in females and those with BMI ≥22.

The TBI-mortality relationship in our study is consistent with a few previous studies. Nonetheless, to the best of our knowledge, this is the largest study with data on TBI in hemodialysis patients. We uniquely observed that the additional prognostic value of low TBI within individuals with normal/high ABI. In addition, this is the first study reporting the prognostic value of TBI from Asia. Since the CVD risk profiles are different between Asians and other racial groups [26], and Asians are the largest racial group migrating to Europe and North America [27], our results may have increasing relevance globally.

Of note, the additional prognostic value of low TBI in normal/high ABI was consistent across key subgroups, although non-significantly more evident in females and patients with higher BMI than their counterparts. Nonetheless, we conducted subgroup analyses without a prespecified hypothesis, and thus the results should be interpreted carefully. Also, we need to acknowledge limited power in this subgroup analysis. Therefore, future larger studies are needed to confirm our observations.

Potential reasons behind the prognostic value of TBI beyond ABI warrant discussion. This may be due to the fact that TBI reflects leg ischemia better than ABI in persons with arterial calcification [28]. Since medial arterial calcification is highly prevalent in the hemodialysis and diabetic population, TBI can be an especially useful prognostic marker in these populations [29]. Another possibility is that, theoretically, TBI can capture arterial stenosis above and below the ankles while ABI reflects only stenosis above the ankles. Thus, TBI may more comprehensively represent overall leg ischemia compared to ABI. However, TBI has been rarely researched in a large dataset, and thus will require further investigation.

Our findings have several important clinical and research implications. Although clinical guidelines currently recommend measuring TBI only in individuals with ABI > 1.4, the independent prognostic value of ABI and TBI indicates the usefulness to simultaneously measure ABI and TBI even among those with normal ABI. The additional time to measure TBI after ABI is < 5 min, and thus it seems worth considering this approach, at least in patients on hemodialysis. As noted above, a few major clinical guidelines recommend 0.7 as the threshold of TBI. However, the evidence behind this recommendation is not necessarily robust. In this context, our results support 0.6 over 0.7 in terms of classifying the mortality risk among persons with normal/high ABI. Interestingly, our observation is consistent with a previous study reporting TBI ≤0.6 as a reasonable threshold to identify hemodialysis patients with PAD [30].

There are some other limitations in our study. First, ABI and TBI were measured during hemodialysis therapy. Hemodynamic changes during hemodialysis therapy may have affected the ABI and TBI measurements. However, the ABI and TBI are determined by the ratios of blood pressure in the ankle and toe, respectively, relative to arm blood pressure, with the blood pressures usually obtained contemporaneously. Therefore, any effect of systemic hemodynamic change on the ABI and TBI is likely to be smaller than the effect on each of brachial, ankle, and toe blood pressures. Second, the analysis is based on a single measurement of ABI and TBI which is prone to non-differential misclassification. Nonetheless, such a misclassification usually biases results towards the null. Third, the measurement of ABI and TBI was based on clinical indication, and thus the generalizability of our results should be carefully evaluated. Fourth, we did not have information on leg vascular procedures and thus cannot infer whether the interpretation of ABI and TBI should be different before and after leg revascularization. Finally, there is the possibility of residual confounding (unmeasured covariates such as parathyroid hormone and triglycerides or unknown confounding).

## Conclusions

In conclusion, among maintenance hemodialysis patients, in contrast to a J-shaped association for ABI, TBI showed a robust and independent dose-response association with mortality. Moreover, TBI classified the risk of mortality in hemodialysis patients with normal/high ABI. Our results support the importance of evaluating TBI in addition to ABI in this clinical population.

## Supplementary information

**Additional file 1.**

## Data Availability

The data that support the findings of this study are available from Nagoya university, but restrictions apply to the availability of these data, which were used under license for the current study, and so are not publicly available. Data are however available from the authors upon reasonable request and with permission of Nagoya university.

## Abbreviations

TBI: Toe-brachial index
ABI: Ankle-brachial index
PAD: Peripheral artery disease
CKD: Chronic kidney disease
CVD: Cardiovascular disease
CAD: Coronary artery disease
HF: Heart failure
HR: Hazard ratios
BMI: Body mass index
IQI: Inter quartile interval
