# Supplementary material for: Additional prognostic value of toe-brachial index beyond ankle-brachial index in hemodialysis patients
Source: BMC Nephrol. 2020 Aug 20;21:353. doi: 10.1186/s12882-020-01991-7 (PMC7439547; doi:10.1186/s12882-020-01991-7)
Supplement: Supplementary file 1 — Additional file 1. [file 12882_2020_1991_MOESM1_ESM.docx]

Figure S1. Flow chart for patient selection.


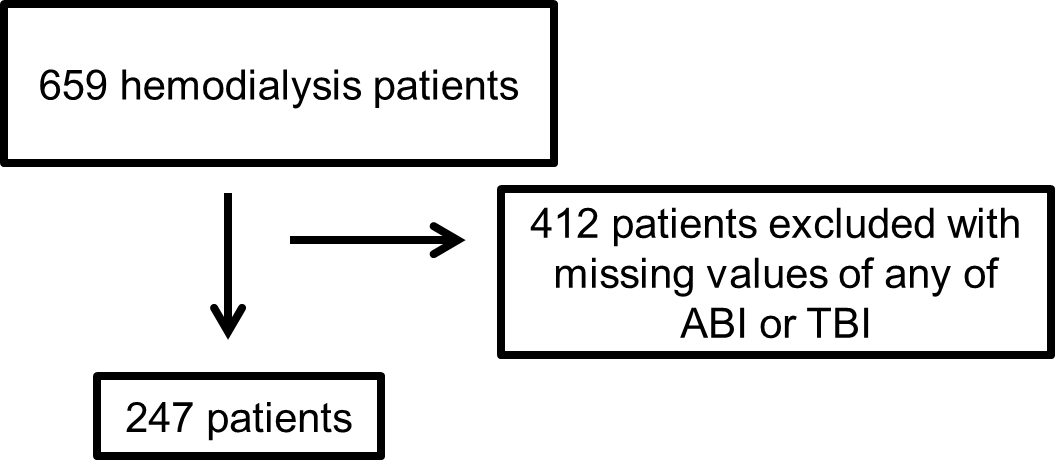


Table S1. Characteristics of study population and excluded population

|  | study population | excluded population |  |
| --- | --- | --- | --- |
| Characteristic | (n=247) | (n=412) | P-value |
| Age at registration (years) | 66.3 ± 12.7 | 66.8 ± 11.6 | 0.59 |
| Hemodialysis vintage (year) | 2.8 ± 5.4 | 9.5 ± 8.2 | <0.01 |
| Gender (male) (%) | 167 (68%) | 289 (70%) | 0.5 |
| Body mass index (kg/m2) | 22.4 ± 3.6 | 22.1 ± 3.6 | 0.58 |
| History of smoking |  |  |  |
| Ever (%) | 51 (21%) | 77 (19%) | 0.54 |
| Never (%) | 161 (65%) | 194 (47%) | <0.01 |
| Unknown (%) | 35 (14%) | 141 (34%) | <0.01 |
| Comorbidity |  |  |  |
| Diabetes mellitus (%) | 115 (48%) | 226 (61%) | <0.01 |
| Hypertension (%) | 173 (75%) | 232 (72%) | 0.49 |
| Lipid metabolism disorder (%) | 26 (12%) | 43 (14%) | 0.36 |
| Peripheral artery disease (%) | 174 (70%) | 137 (33%) | <0.01 |
| Other CVDs (CAD, HF, stroke) (%) | 162 (66%) | 240 (58%) | 0.06 |
| Primary disease |  |  |  |
| Diabetic nephropathy (%) | 111 (45%) | 208 (50%) | 0.17 |
| Nephrosclerosis (%) | 16 (6%) | 49 (12%) | 0.02 |
| Chronic glomerulonephritis (%) | 65 (26%) | 55 (13%) | <0.01 |
| Polycystic kidney disease (%) | 10 (4%) | 14 (3%) | 0.67 |
| Other (%) | 23 (9%) | 29 (7%) | 0.29 |
| Unknown (%) | 22 (9%) | 57 (14%) | 0.06 |
| Systolic blood pressure (mmHg) | 145.7 ± 23.1 | 148.6 ± 23.0 | 0.44 |
| Diastolic blood pressure (mmHg) | 79.8 ± 14.9 | 80.8 ± 12.4 | 0.63 |
| Total cholesterol (mg/dl) | 159.0 ± 35.7 | 155.6 ± 33.4 | 0.28 |
| Oral medication |  |  |  |
| Antihypertensive drugs (%) | 149 (%) | 263 (%) | 0.37 |
| Lipid-lowering drugs (%) | 8 (3%) | 78 (19%) | <0.01 |
| Hemoglobin (g/dl) | 10.7 ± 1.3 | 10.7 ± 1.3 | 0.99 |
| Albumin (g/dl) | 3.6 ± 0.4 | 3.7 ± 0.3 | <0.01 |
| Calcium (mg/dl) | 8.6 ± 0.7 | 8.8 ± 0.7 | 0.04 |
| Phosphate (mg/dl) | 4.5 ± 1.9 | 5.1 ± 1.4 | <0.01 |

Values are mean ± SD, % of the total. PAD peripheral artery disease, CVD cardiovascular disease, CAD coronary artery disease, HF heart failure

Table S2. Baseline characteristics of patients stratified by ABI quartile

|  | Lower ABI (Quartile) | | | |  |  |  |
| --- | --- | --- | --- | --- | --- | --- | --- |
|  | Q1 (0.31-0.91) | Q2 (0.92-1.07) | Q3 (1.08-1.17) | Q4 (1.18-1.41) | Total |  |  |
| Characteristic | (n=62) | (n=66) | (n=58) | (n=61) | (n=247) | P-value | Post hoc |
| Age at registration (years) | 69.5 ± 12.2 | 66.3 ± 12.5 | 67.8 ± 10.0 | 63.7 ± 11.0 | 66.8 ± 11.6 | <0.05 | c |
| Dialysis vintage (years) | 6.3 ± 6.3 | 10.6 ± 8.6 | 10.6 ± 8.6 | 10.5 ± 8.3 | 9.5 ± 8.2 | <0.01 | a, b, c |
| Gender (male) | 42 (68%) | 40 (61%) | 36 (62%) | 49 (80%) | 167 (68%) | 0.079 |  |
| Body mass index (kg/m^2^) | 22.4 ± 3.7 | 22.3 ± 3.8 | 21.5 ± 2.7 | 22.1 ± 4.1 | 22.1 ± 3.6 | 0.505 |  |
| History of smoking |  |  |  |  |  |  |  |
| Ever | 11 (18%) | 16 (24%) | 11 (19%) | 13 (21%) | 51 (21%) | 0.815 |  |
| Never | 41 (66%) | 37 (56%) | 41 (71%) | 42 (69%) | 161 (65%) | 0.314 |  |
| Unknown | 10 (16%) | 13 (20%) | 6 (10%) | 6 (10%) | 35 (14%) | 0.325 |  |
| Comorbidity |  |  |  |  |  |  |  |
| Diabetes mellitus | 43 (69%) | 28 (44%) | 21 (38%) | 23 (38%) | 115 (48%) | <0.01 | a, b, c |
| Hypertension | 40 (68%) | 46 (75%) | 41 (77%) | 46 (79%) | 173 (75%) | 0.505 |  |
| Lipid metabolism disorder | 11 (19%) | 5 (8%) | 7 (13%) | 3 (5%) | 26 (12%) | 0.116 |  |
| PAD | 53 (85%) | 52 (79%) | 33 (57%) | 36 (59%) | 174 (70%) | <0.01 | b, c, d |
| Other CVDs (CAD, HF, stroke) | 51 (82%) | 47 (71%) | 36 (62%) | 28 (46%) | 162 (66%) | <0.01 | c, e |
| Primary disease |  |  |  |  |  |  |  |
| Diabetic nephropathy | 42 (68%) | 27 (41%) | 20 (34%) | 22 (36%) | 111 (45%) | <0.01 | a, b, c |
| Nephrosclerosis | 4 (6%) | 4 (6%) | 3 (5%) | 5 (8%) | 16 (6%) | 0.925 |  |
| Chronic glomerulonephritis | 10 (16%) | 18 (27%) | 22 (38%) | 15 (25%) | 65 (26%) | 0.058 |  |
| Polycystic kidney disease | 2 (3%) | 2 (3%) | 5 (9%) | 1 (2%) | 10 (4%) | 0.231 |  |
| Other | 1 (2%) | 8 (12%) | 5 (9%) | 9 (15%) | 23 (9%) | 0.067 |  |
| Unknown | 3 (5%) | 7 (11%) | 3 (5%) | 9 (15%) | 22 (9%) | 0.168 |  |
| Systolic blood pressure (mmHg) | 156 ± 23 | 143 ± 26 | 150 ± 23 | 145 ± 18 | 149 ± 23 | <0.01 | a, c |
| Diastolic blood pressure (mmHg) | 80 ± 11 | 79 ± 14 | 83 ± 11 | 82 ± 12 | 81 ± 12 | 0.268 |  |
| Total cholesterol (mg/dl) | 164 ± 34 | 160 ± 35 | 153 ± 33 | 144 ± 29 | 156 ± 33 | <0.01 | c |
| Oral medication |  |  |  |  |  |  |  |
| Antihypertensive drugs | 42 (68%) | 36 (55%) | 30 (52%) | 41 (67%) | 149 (60%) | 0.149 |  |
| Lipid-lowering drugs | 3 (5%) | 1 (2%) | 2 (3%) | 2 (3%) | 8 (3%) | 0.771 |  |
| Hemoglobin (g/dl) | 10.4 ± 1.1 | 10.9 ± 1.5 | 10.8 ± 1.2 | 10.7 ± 1.3 | 10.7 ± 1.3 | 0.159 |  |
| Albumin (g/dl) | 3.6 ± 0.3 | 3.7 ± 0.3 | 3.7 ± 0.3 | 3.8 ± 0.3 | 3.7 ± 0.3 | <0.05 | c |
| Calcium (mg/dl) | 8.6 ± 0.6 | 8.6 ± 0.7 | 8.8 ± 0.8 | 9.0 ± 0.6 | 8.8 ± 0.7 | <0.01 | c, e |
| Phosphate (mg/dl) | 4.9 ± 1.7 | 4.9 ± 1.2 | 5.2 ± 1.5 | 5.4 ± 1.4 | 5.1 ± 1.4 | 0.184 |  |
| TBI | 0.47 ± 0.13 | 0.62 ± 0.13 | 0.71 ± 0.14 | 0.73 ± 0.19 | 0.63 ± 0.18 | <0.01 | a, b, c, d, e, f |

Values are mean ± SD, % of the total. Missing values: Body mass index (n=3), History of smoking (n=35), Diabetes mellitus (n=6), Hypertension (n=16), Lipid metabolism disorder (n=21), Systolic blood pressure (n=2), Total cholesterol (n=29), Hemoglobin (n=23), Albumin (n=34), Calcium (n=8), and Phosphate (n=8). PAD peripheral artery disease, CVD cardiovascular disease, CAD coronary artery disease, HF heart failure, TBI toe-brachial index ^a^significant difference between Q1 vs. Q2; ^b^significant difference between Q1 vs. Q3; ^c^significant difference between Q1 vs. Q4; ^d^significant difference between Q2 vs. Q3; ^e^significant difference between Q2 vs. Q4; ^f^significant difference between Q3 vs. Q4.

Figure S2. Survival estimates (CVD mortality [upper]; non-CVD mortality [lower]) according to ABI and TBI quartiles.


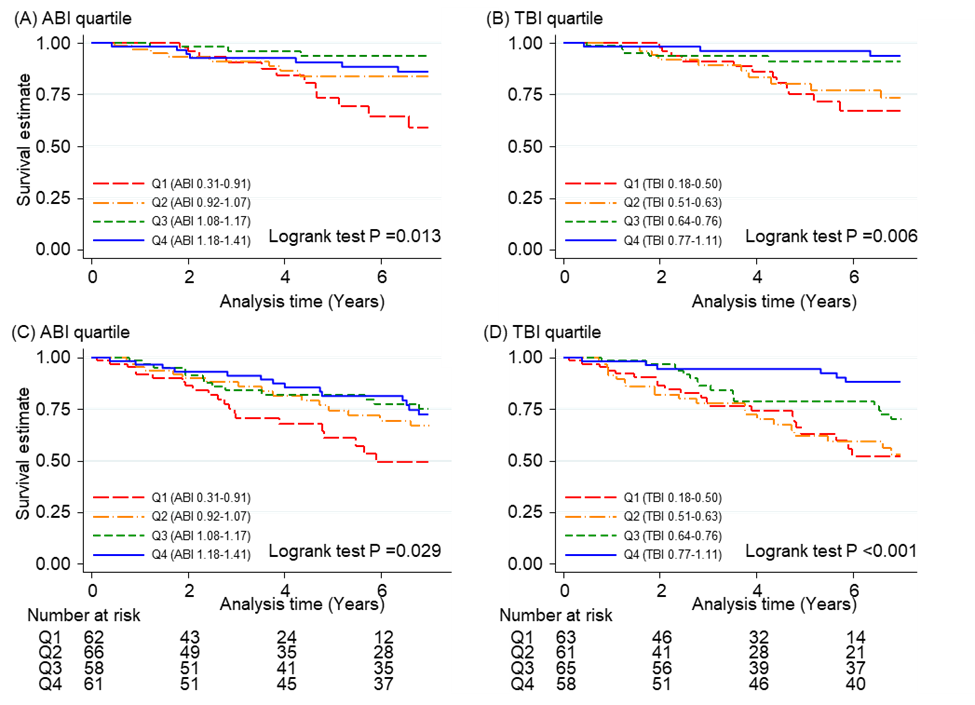


Table S3. HRs (95% CI) of CVD and non-CVD mortality according to ABI and TBI quartile

| ABI | Q1 (0.31-0.91) | Q2 (0.92-1.07) | Q3 (1.08-1.17) | Q4 (1.18-1.41) | P for trend (Q1-3) |
| --- | --- | --- | --- | --- | --- |
| CVD mortality | 12 deaths / 62 | 8 deaths / 66 | 3 deaths / 58 | 7 deaths / 61 | (n=186) |
| Model 1 | 5.87 (1.65-20.90) ** | 2.92 (0.78-11.03) | ref | 2.21 (0.57-8.56) | 0.003 |
| Model 2 | 5.77 (1.61-20.68) ** | 2.96 (0.78-11.18) | ref | 2.62 (0.67-10.23) | 0.007 |
| Model 3 | 4.88 (1.31-18.22) * | 2.69 (0.71-10.26) | ref | 2.65 (0.68-10.34) | 0.014 |
| Model 4 | 5.03 (1.28-19.77) * | 2.95 (0.74-11.74) | ref | 2.41 (0.60-9.59) | 0.017 |
| non-CVD mortality | 21 deaths / 62 | 16 deaths / 66 | 13 deaths / 58 | 14 deaths / 61 | (n=186) |
| Model 1 | 2.40 (1.19-4.82) * | 1.35 (0.65-2.81) | ref | 1.04 (0.49-2.20) | 0.001 |
| Model 2 | 2.19 (1.08-4.45) * | 1.30 (0.62-2.71) | ref | 1.36 (0.63-2.92) | 0.008 |
| Model 3 | 2.03 (0.97-4.23) | 1.13 (0.53-2.39) | ref | 1.34 (0.62-2.87) | 0.012 |
| Model 4 | 1.89 (0.88-4.09) ** | 1.07 (0.49-2.33) | ref | 1.40 (0.64-3.06) | 0.02 |
| TBI | Q1 (0.18-0.50) | Q2 (0.51-0.63) | Q3 (0.64-0.76) | Q4 (0.77-1.11) | P for trend (Q1-4) |
| CVD mortality | 12 deaths / 63 | 10 deaths / 61 | 5 deaths / 65 | 3 deaths / 58 | (n=247) |
| Model 1 | 5.48 (1.54-19.50) ** | 4.86 (1.33-17.71) * | 1.74 (0.41-7.27) | ref | <0.001 |
| Model 2 | 4.24 (1.16-15.40) * | 4.12 (1.12-15.16) * | 1.44 (0.34-6.10) | ref | 0.003 |
| Model 3 | 2.84 (0.74-10.83) | 2.90 (0.76-11.03) | 1.04 (0.24-4.60) | ref | 0.029 |
| Model 4 | 3.63 (0.95-13.87) | 3.51 (0.91-13.59) | 1.33 (0.30-5.85) | ref | 0.029 |
| non-CVD mortality | 21 deaths / 63 | 21 deaths / 61 | 16 deaths / 65 | 5 deaths / 58 | (n=247) |
| Model 1 | 4.92 (1.98-12.24) ** | 5.09 (2.05-12.63) ** | 2.74 (1.07-7.00) * | ref | <0.001 |
| Model 2 | 2.96 (1.18-7.47) * | 3.95 (1.58-9.88) ** | 1.86 (0.72-4.80) | ref | 0.03 |
| Model 3 | 3.30 (1.24-8.83) * | 4.32 (1.62-11.56) ** | 2.09 (0.76-5.73) | ref | 0.043 |
| Model 4 | 3.13 (1.16-8.40) * | 4.46(1.67-11.96) ** | 2.26 (0.83-6.16) | ref | 0.065 |

Model 1; unadjusted

Model 2; adjusted for age and gender

Model 3; Model 2+ diabetes mellitus, smoking status, history of cardiovascular disease, and hemodialysis vintage

Model 4; Model 3+ systolic blood pressure, total cholesterol, hemoglobin, albumin, calcium, and phosphate

* P < 0.05, ** P < 0.01

Table S4. HRs (95% CI) of all-cause mortality according to ABI/TBI quartile in patients with ABI <1.4.

| ABI | Q1 (0.31-0.91) | Q2 (0.92-1.07) | Q3 (1.08-1.17) | Q4 (1.18-1.41) | P for trend (Q1-3) |
| --- | --- | --- | --- | --- | --- |
| All-cause mortality | 41 deaths / 62 | 26 deaths / 57 | 21 deaths / 65 | 20 deaths / 51 | (n=184) |
| Model 1 | 3.13 (1.85-5.32) ** | 1.75 (0.99-3.12) | ref | 1.17 (0.63-2.15) | <0.001 |
| Model 2 | 2.89 (1.69-4.94) ** | 1.61 (0.91-2.87) | ref | 1.36 (0.73-2.54) | 0.008 |
| Model 3 | 2.74 (1.56-4.79) ** | 1.47 (0.82-2.65) | ref | 1.42 (0.76-2.63) | 0.012 |
| Model 4 | 2.77 (1.53-4.99) ** | 1.61 (0.88-2.96) | ref | 1.48 (0.78-2.78) | 0.019 |
| TBI | Q1 (0.18-0.50) | Q2 (0.51-0.63) | Q3 (0.64-0.76) | Q4 (0.77-1.11) | P for trend (Q1-4) |
| All-cause mortality | 38 deaths / 61 | 34 deaths / 59 | 23 deaths / 59 | 13 deaths / 56 | (n=235) |
| Model 1 | 4.02 (2.14-7.57) ** | 3.80 (2.00-7.22) ** | 1.97 (1.00-3.90) | ref | <0.001 |
| Model 2 | 2.83 (1.49-5.40) ** | 3.21 (1.68-6.13) ** | 1.55 (0.78-3.08) | ref | 0.001 |
| Model 3 | 2.43 (1.23-4.81) ** | 2.79 (1.41-5.52) ** | 1.32 (0.64-2.72) | ref | 0.01 |
| Model 4 | 2.51 (1.27-4.99) ** | 2.91 (1.47-5.76) ** | 1.46 (0.71-3.00) | ref | 0.011 |

Model 1; unadjusted

Model 2; adjusted for age and gender

Model 3; Model 2+ diabetes mellitus, smoking status, history of cardiovascular disease, and hemodialysis vintage

Model 4; Model 3+ systolic blood pressure, total cholesterol, hemoglobin, albumin, calcium, and phosphate

* P < 0.05, ** P < 0.01

Figure S3. Survival estimates (CVD mortality [upper]; non-CVD mortality [lower]) according to three categories of ABI/TBI.


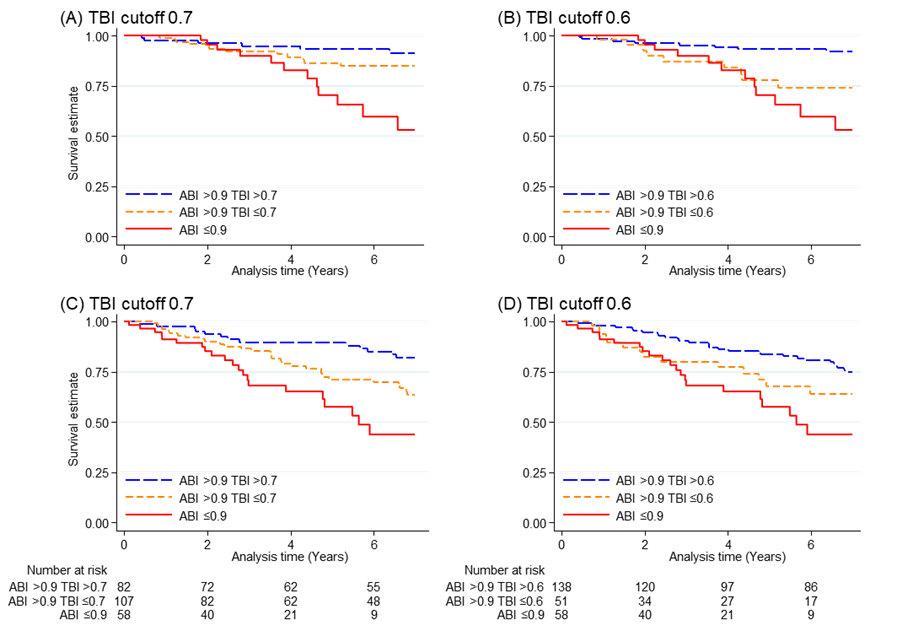


Table S5. HRs (95% CI) of CVD and non-CVD mortality according to three categories with different cutoffs.

|  | ABI >0.9 TBI >0.7 | ABI >0.9 TBI ≤0.7 | ABI ≤0.9 |
| --- | --- | --- | --- |
| CVD mortality | 6 deaths / 82 | 12 deaths / 107 | 12 deaths / 58 |
| Model 1 | ref | 1.91 (0.72-5.10) | 4.88 (1.81-13.15) ** |
| Model 2 | ref | 1.64 (0.61-4.41) | 3.93 (1.43-10.84) ** |
| Model 3 | ref | 1.23 (0.45-3.41) | 2.91 (0.98-8.63) * |
| Model 4 | ref | 1.56 (0.55-4.40) | 3.52 (1.13-10.90) * |
| non-CVD mortality | 13 deaths / 82 | 30 deaths / 107 | 21 deaths / 58 |
| Model 1 | ref | 2.20 (1.15-4.22) * | 3.97 (1.97-8.00) ** |
| Model 2 | ref | 1.62 (0.84-3.15) | 2.60 (1.26-5.36) ** |
| Model 3 | ref | 1.61 (0.80-3.25) | 2.63 (1.21-5.71) * |
| Model 4 | ref | 1.75 (0.86-3.56) | 2.65 (1.18-5.96) * |
|  | ABI >0.9 TBI >0.6 | ABI >0.9 TBI ≤0.6 | ABI ≤0.9 |
| CVD mortality | 9 deaths / 138 | 9 deaths / 51 | 12 deaths / 58 |
| Model 1 | ref | 3.66 (1.45-9.25) ** | 5.26 (2.19-12.61) ** |
| Model 2 | ref | 3.50 (1.38-8.85) ** | 4.57 (1.87-11.16) ** |
| Model 3 | ref | 2.86 (1.11-7.36) * | 3.91 (1.52-10.08) ** |
| Model 4 | ref | 3.01 (1.14-7.96) * | 4.14 (1.50-11.42) ** |
| non-CVD mortality | 29 deaths / 138 | 14 deaths / 51 | 21 deaths / 58 |
| Model 1 | ref | 1.79 (0.94-3.39) | 2.87 (1.62-5.08) ** |
| Model 2 | ref | 1.65 (0.87-3.15) | 2.17 (1.21-3.92) ** |
| Model 3 | ref | 1.62 (0.84-3.16) | 2.20 (1.18-4.11) * |
| Model 4 | ref | 1.44 (0.73-2.85) | 2.01 (1.04-3.89) * |

Model 1; unadjusted

Model 2; adjusted for age and gender

Model 3; Model 2+ diabetes mellitus, smoking status, history of cardiovascular disease, and hemodialysis vintage

Model 4; Model 3+ systolic blood pressure, total cholesterol, hemoglobin, albumin, calcium, and phosphate

* P < 0.05, ** P < 0.01

Table S6. HRs (95% CI) of all-cause mortality according to three categories in patients with ABI <1.4.

|  | ABI >0.9 TBI >0.7 | ABI >0.9 TBI ≤0.7 | ABI ≤0.9 |
| --- | --- | --- | --- |
| All-cause mortality | 22 deaths / 78 | 46 deaths / 99 | 40 deaths / 58 |
| Model 1 | ref | 2.09 (1.26-3.47) ** | 4.21 (2.49-7.13) ** |
| Model 2 | ref | 1.71 (1.02-2.87) * | 3.19 (1.86-5.47) ** |
| Model 3 | ref | 1.51 (0.88-2.59) | 2.91 (1.63-5.19) ** |
| Model 4 | ref | 1.67 (0.97-2.87) | 2.98 (1.64-5.42) ** |
|  | ABI >0.9 TBI >0.6 | ABI >0.9 TBI ≤0.6 | ABI ≤0.9 |
| All-cause mortality | 43 deaths / 129 | 25 deaths / 48 | 40 deaths / 58 |
| Model 1 | ref | 2.07 (1.26-3.40) ** | 3.37 (2.17-5.21) ** |
| Model 2 | ref | 1.91 (1.16-3.13) * | 2.77 (1.77-4.33) ** |
| Model 3 | ref | 1.76 (1.06-2.94) * | 2.68 (1.67-4.31) ** |
| Model 4 | ref | 1.71 (1.01-2.87) * | 2.54 (1.54-4.18) ** |

Model 1; unadjusted

Model 2; adjusted for age and gender

Model 3; Model 2+ diabetes mellitus, smoking status, history of cardiovascular disease, and hemodialysis vintage

Model 4; Model 3+ systolic blood pressure, total cholesterol, hemoglobin, albumin, calcium, and phosphate

* P < 0.05, ** P < 0.01

Table S7. HRs (95% CI) of all-cause mortality according to ABI and TBI measured within two years.

| ABI | Q1 (0.31-0.88) | Q2 (0.89-1.06) | Q3 (1.07-1.16) | Q4 (1.17-1.41) | P for trend (Q1-3) |
| --- | --- | --- | --- | --- | --- |
| All-cause mortality | 36 deaths / 51 | 22 deaths / 49 | 12 deaths / 47 | 23 deaths / 46 | (n=147) |
| Model 1 | 4.84 (2.50-9.35) ** | 2.26 (1.12-4.57) * | ref | 2.21 (1.10-4.45) * | <0.001 |
| Model 2 | 4.03 (2.07-7.84) ** | 2.03 (0.99-4.13) | ref | 2.07 (1.03-4.17) * | 0.002 |
| Model 3 | 3.98 (1.97-8.07) ** | 1.94 (0.94-4.02) | ref | 2.08 (1.02-4.24) * | 0.002 |
| Model 4 | 3.85 (1.87-7.93) ** | 2.06 (0.98-4.32) | ref | 2.09 (1.01-4.31) * | 0.003 |
| TBI | Q1 (0.18-0.50) | Q2 (0.51-0.63) | Q3 (0.64-0.75) | Q4 (0.76-1.10) | P for trend (Q1-4) |
| All-cause mortality | 36 deaths / 53 | 27 deaths / 50 | 17 deaths / 44 | 13 deaths / 46 | (n=193) |
| Model 1 | 3.51 (1.85-6.65) ** | 2.77 (1.43-5.38) ** | 1.48 (0.72-3.06) | ref | <0.001 |
| Model 2 | 2.68 (1.40-5.11) ** | 2.49 (1.28-4.85) ** | 1.27 (0.61-2.64) | ref | 0.001 |
| Model 3 | 2.85 (1.42-5.71) ** | 2.62 (1.30-5.29) ** | 1.41 (0.66-3.04) | ref | 0.001 |
| Model 4 | 3.14 (1.56-6.32) ** | 2.76 (1.35-5.61) ** | 1.51 (0.70-3.27) | ref | 0.001 |

Model 1; unadjusted

Model 2; adjusted for age and gender

Model 3; Model 2+ diabetes mellitus, smoking status, history of cardiovascular disease, and hemodialysis vintage

Model 4; Model 3+ systolic blood pressure, total cholesterol, hemoglobin, albumin, calcium, and phosphate

* P < 0.05, ** P < 0.01

Table S8. HRs (95% CI) of all-cause mortality across three categories of ABI/TBI measured within two years.

|  | ABI >0.9 TBI >0.7 | ABI >0.9 TBI ≤0.7 | ABI ≤0.9 |
| --- | --- | --- | --- |
| All-cause mortality | 18 deaths / 58 | 38 deaths / 83 | 37 deaths / 52 |
| Model 1 | ref | 1.71 (0.97-2.99) | 3.90 (2.20-6.90) ** |
| Model 2 | ref | 1.41 (0.80-2.49) | 3.02 (1.69-5.40) ** |
| Model 3 | ref | 1.44 (0.79-2.63) | 3.06 (1.63-5.75) ** |
| Model 4 | ref | 1.72 (0.94-3.15) | 3.23 (1.70-6.13) ** |
|  | ABI >0.9 TBI >0.6 | ABI >0.9 TBI ≤0.6 | ABI ≤0.9 |
| All-cause mortality | 37 deaths / 104 | 19 deaths / 37 | 37 deaths / 52 |
| Model 1 | ref | 1.87 (1.08-3.26) * | 3.33 (2.10-5.30) ** |
| Model 2 | ref | 1.84 (1.05-3.21) * | 2.88 (1.80-4.61) ** |
| Model 3 | ref | 1.87 (1.05-3.34) * | 2.89 (1.75-4.78) ** |
| Model 4 | ref | 1.96 (1.08-3.58) * | 2.77 (1.64-4.68) ** |

Model 1; unadjusted

Model 2; adjusted for age and gender

Model 3; Model 2+ diabetes mellitus, smoking status, history of cardiovascular disease, and hemodialysis vintage

Model 4; Model 3+ systolic blood pressure, total cholesterol, hemoglobin, albumin, calcium, and phosphate

* P < 0.05, ** P < 0.01

Table S9. HRs (95% CI) of all-cause mortality according to ABI and TBI in complete case analysis.

| ABI | Q1 (0.31-0.91) | Q2 (0.92-1.07) | Q3 (1.08-1.17) | Q4 (1.18-1.41) | P for trend (Q1-3) |
| --- | --- | --- | --- | --- | --- |
| Model 1, 116 deaths/247 | 41 deaths / 62 | 29 deaths / 66 | 19 deaths / 58 | 27 deaths / 61 | (n=186) |
| HR (95% CI) | 3.15 (1.82-5.44) ** | 1.67 (0.94-2.98) | ref | 1.36 (0.76-2.45) | <0.001 |
| Model 2, 116 deaths/247 | 41 deaths / 62 | 29 deaths / 66 | 19 deaths / 58 | 27 deaths / 61 | (n=186) |
| HR (95% CI) | 2.99 (1.72-5.19) ** | 1.69 (0.94-3.01) | ref | 1.62 (0.90-2.93) | <0.001 |
| Model 3, 98 deaths/212 | 36 deaths / 52 | 21 deaths / 53 | 18 deaths / 52 | 23 deaths / 55 | (n=157) |
| HR (95% CI) | 2.77 (1.52-5.04) ** | 1.18 (0.62-2.24) | ref | 1.42 (0.76-2.64) | <0.001 |
| Model 4, 83 deaths/176 | 32 deaths / 47 | 16 deaths / 43 | 13 deaths / 39 | 22 deaths / 47 | (n=129) |
| HR (95% CI) | 2.96 (1.47-5.94) ** | 0.96 (0.44-2.07) | ref | 1.48 (0.73-3.01) | <0.001 |
| TBI | Q1 (0.18-0.50) | Q2 (0.51-0.63) | Q3 (0.64-0.76) | Q4 (0.77-1.11) | P for trend (Q1-4) |
| Model 1, 116 deaths/247 | 40 deaths / 63 | 35 deaths / 61 | 27 deaths / 65 | 14 deaths / 58 | (n=247) |
| HR (95% CI) | 3.91 (2.12-7.21) ** | 3.60 (1.93-6.70) ** | 1.98 (1.04-3.78) * | ref | <0.001 |
| Model 2, 116 deaths/247 | 40 deaths / 63 | 35 deaths / 61 | 27 deaths / 65 | 14 deaths / 58 | (n=247) |
| HR (95% CI) | 2.74 (1.47-5.11) ** | 3.01 (1.61-5.63) ** | 1.56 (0.81-2.99) | ref | <0.001 |
| Model 3, 98 deaths/212 | 33 deaths / 53 | 30 deaths / 50 | 23 deaths / 59 | 12 deaths / 50 | (n=212) |
| HR (95% CI) | 2.53 (1.24-5.16) * | 3.12 (1.53-6.37) ** | 1.39 (0.66-2.92) | ref | 0.013 |
| Model 4, 83 deaths/176 | 31 deaths / 50 | 24 deaths / 41 | 20 deaths / 49 | 11 deaths / 40 | (n=176) |
| HR (95% CI) | 2.31 (1.09-4.87) * | 2.21 (1.03-4.76) * | 1.30 (0.60-2.81) | ref | 0.050 |

Model 1; unadjusted

Model 2; adjusted for age and gender

Model 3; Model 2+ diabetes mellitus, smoking status, history of cardiovascular disease, and hemodialysis vintage

Model 4; Model 3+ systolic blood pressure, total cholesterol, hemoglobin, albumin, calcium, and phosphate

* P < 0.05, ** P < 0.01

Table S10. HRs (95% CI) of all-cause mortality according to three categories in complete case analysis.

|  | ABI >0.9 TBI >0.7 | ABI >0.9 TBI ≤0.7 | ABI ≤0.9 |
| --- | --- | --- | --- |
| Model 1, 116 deaths / 247 | 25 deaths / 82 | 51 deaths / 107 | 40 deaths / 58 |
| HR (95% CI) | ref | 1.94 (1.20-3.13) ** | 3.84 (2.32-6.37) ** |
| Model 2, 116 deaths / 247 | 25 deaths / 82 | 51 deaths / 107 | 40 deaths / 58 |
| HR (95% CI) | ref | 1.60 (0.99-2.61) | 2.89 (1.72-4.86) ** |
| Model 3, 98 deaths / 212 | 21 deaths / 70 | 42 deaths / 92 | 35 deaths / 50 |
| HR (95% CI) | ref | 1.49 (0.85-2.60) | 3.09 (1.68-5.66) ** |
| Model 4, 83 deaths / 176 | 20 deaths / 56 | 32 deaths / 75 | 31 deaths / 45 |
| HR (95% CI) | ref | 1.14 (0.63-2.05) | 2.70 (1.42-5.13) ** |
|  | ABI >0.9 TBI >0.6 | ABI >0.9 TBI ≤0.6 | ABI ≤0.9 |
| Model 1, 116 deaths / 247 | 48 deaths / 138 | 28 deaths / 51 | 40 deaths / 58 |
| HR (95% CI) | ref | 2.13 (1.34-3.41) ** | 3.23 (2.11-4.95) ** |
| Model 2, 116 deaths / 247 | 48 deaths / 138 | 28 deaths / 51 | 40 deaths / 58 |
| HR (95% CI) | ref | 1.97 (1.23-3.15) ** | 2.64 (1.71-4.08) ** |
| Model 3, 98 deaths / 212 | 40 deaths / 119 | 23 deaths / 43 | 35 deaths / 50 |
| HR (95% CI) | ref | 1.98 (1.16-3.36) * | 2.97 (1.80-4.90) ** |
| Model 4, 83 deaths / 176 | 34 deaths / 96 | 18 deaths / 35 | 31 deaths / 45 |
| HR (95% CI) | ref | 1.64 (0.90-3.00) | 2.95 (1.67-5.21) ** |

Model 1; unadjusted

Model 2; adjusted for age and gender

Model 3; Model 2+ diabetes mellitus, smoking status, history of cardiovascular disease, and hemodialysis vintage

Model 4; Model 3+ systolic blood pressure, total cholesterol, hemoglobin, albumin, calcium, and phosphate

* P < 0.05, ** P < 0.01
